# Characterization and Correlation of Microbiota and Higher Alcohols Based on Metagenomic and Metabolite Profiling during Rice-Flavor Baijiu Fermentation

**DOI:** 10.3390/foods12142720

**Published:** 2023-07-16

**Authors:** Hong Wang, Minqian Zhang, Chunyun Qu, Yongtao Fei, Jinglong Liang, Weidong Bai, Wenhong Zhao, Gengsheng Xiao, Gongliang Liu

**Affiliations:** 1 Guangdong Provincial Key Laboratory of Lingnan Specialty Food Science and Technology, College of Light Industry and Food Technology, Zhongkai University of Agriculture and Engineering, Guangzhou 510225, China; whongaq@163.com (H.W.); gshxiao@aliyun.com (G.X.); 2 Key Laboratory of Green Processing and Intelligent Manufacturing of Lingnan Specialty Food, Ministry of Agriculture and Rural Affairs, Zhongkai University of Agriculture and Engineering, Guangzhou 510225, China; 3 Academy of Contemporary Agricultural Engineering Innovations, Zhongkai University of Agriculture and Engineering, Guangzhou 510225, China

**Keywords:** higher alcohol precursors, metabolic pathways, capillary gas chromatography, metagenome sequencing

## Abstract

Higher alcohol, as an inevitable product of fermentation, plays an important role in the flavor and quality of Baijiu. However, the relationship between the complex microbial metabolism and the formation of higher alcohols in rice-flavor Baijiu was not clear. To investigate the relationship between microorganisms and higher alcohol production, two fermentation mashes inoculated with starters from Heyuan Jinhuangtian Liquor Co., Ltd. (Heyuan, China) as JM and Guangdong Changleshao Co., Ltd. (Meizhou, China) as CM, respectively, with significant differences in higher alcohol profiles during rice-flavor Baijiu fermentation were selected. In general, higher alcohols presented a rapid accumulation during the early fermentation stages, especially in JM, with higher and faster increases than those in CM. As for their precursors including amino acids, pyruvic acid and ketoacids, complex variations were observed during the fermentation. Metagenomic results indicated that *Saccharomyces cerevisiae* and *Rhizopus microsporus* were the microorganisms present throughout the brewing process in JM and CM, and the relative abundance of *R. microsporus* in JM was significantly higher than that in CM. The results of higher alcohol metabolism in JM may contribute to the regulation of higher alcohols in rice-flavor Baijiu.

## 1. Introduction

Rice-flavor Baijiu is one of the four dominant aroma types of Chinese Baijiu, which is produced from rice by semi-solid fermentation with Xiaoqu as the fermentation starter. The characteristic flavor compounds in rice-flavor Baijiu are ethyl acetate, ethyl lactate and β-phenylethanol, which give it a soft sweetness and clean aftertaste [1]. Compared with the other three dominant aroma types of Chinese Baijiu (strong-, sauce- and light-flavor), the content of flavor components in rice-flavor Baijiu is lower, but the content of higher alcohols (such as isobutanol, isoamyl alcohol and β-phenylethanol) is higher [2,3]. Higher alcohols are a group of by-products of alcoholic fermentation with more than two carbons, which is inevitable in Baijiu brewing. The composition and content of higher alcohols play an important role in the aroma and quality of Baijiu [3]. A moderate amount of these compounds contributes to the mellow and sweet taste of Baijiu. However, excessive amounts of higher alcohols will not only destroy the flavor of Baijiu, but also cause headache and hangover after drinking [4,5,6].

The main higher alcohols in Baijiu include n-propanol, isobutanol, isoamyl alcohol, 2-methylbutanol and β-phenylethanol, which are the main metabolites of *Saccharomyces cerevisiae* [7]. Studies have shown that the Ehrlich and Harris pathways are the two main metabolic pathways of higher alcohols [8,9]. The mechanism of the Ehrlich pathway is to catalyze the transamination of branched-chain amino acids, such as valine, leucine and isoleucine to produce the corresponding higher alcohols by decarboxylation and dehydrogenation of 2-oxoisovalarate, 4-methyl-2-oxopentanoate and 3-methyl-2-oxopentanoate, respectively [10]. The Harris metabolic pathway mainly produces higher alcohols by catalysis of pyruvate with lactate acetylase, decarboxylase and dehydrogenase [11,12].

Yeast, especially *S. cerevisiae*, is considered to be the main microorganism that is responsible for the formation of higher alcohols in brewing because of its various active enzymes involved in the Ehrlich and amino metabolic pathways, such as 2-ketoacid decarboxylase (KDC), alcohol dehydrogenase (ADH6), phenylpyruvate decarboxylase (ARO10), branched-chain amino acid transaminases (BAT1 and BAT2), acetolactate synthase (ILV2), dihydroxyacid dehydratase (ILV3) and acetohydroxyacid reductoisomerase (ILV5) [13,14]. In addition, other microbial communities such as non-*Saccharomyces* yeasts, molds and bacteria have been reported to be involved in higher alcohol production. Except for single species of microorganisms, their synergistic effect leads to the complexity of higher alcohol production [15,16].

The process of microbial growth and metabolite accumulation is at basis of Baijiu brewing, and the composition and accumulation of higher alcohols in Baijiu are directly related to the synergistic interaction between populations [17]. Compared with strong- and light-flavor Baijiu, there are fewer microbial species and dominating microorganisms prominent in the fermentation process of rice-flavor Baijiu, which may be the primary cause of the lack of a wide range of flavor compounds in rice-flavor Baijiu [2]. In addition, nutrients in rice cultivars significantly influence the higher alcohol accumulation, especially in the earlier stage of rice wine fermentation [6]. As for another traditional Chinese alcoholic beverage, a total of 684 correlations between higher alcohols and microorganisms were investigated in northern Huangjiu fermentation [18]. Nevertheless, the relationship of microbial succession, the profile of higher alcohols and their precursors is not fully understood, especially for rice-flavor Baijiu fermentation.

In this study, two types of fermented mash with significantly different higher alcohol production were selected to investigate the correlation between brewing microorganisms and higher alcohol contents. The contents of higher alcohols and related precursors in the fermentation of rice-flavor Baijiu were detected dynamically. The microbial community structure of two mashes from different fermentation periods was analyzed by metagenomic sequencing. The relationship between the key microorganisms and higher alcohols was identified, and the period of higher alcohol formation was clarified, which could contribute to control the content of higher alcohols and improve the taste and quality of rice-flavor Baijiu.

## 2. Materials and Methods

### 2.1. Sample Collection

**Figure 1:**
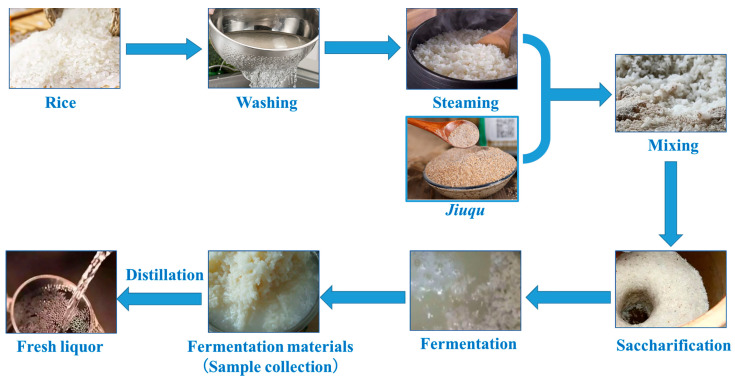
Fermentation process of rice-flavor Baijiu.

Two fermented mashes selected in this study were inoculated, respectively, with Jinhuangtian Jiuqu (known as fermented starter) from Heyuan Jinhuangtian Liquor Co., Ltd. (Heyuan, China) as JM and Changleshao Jiuqu from Guangdong Changleshao Co., Ltd. (Meizhou, China) as CM. Based on our previous results (Figure S1), the content of higher alcohols in JM was much higher than that in CM. The fermentation process of rice-flavor Baijiu was carried out as shown in Figure 1. In general, Japonica rice was washed and soaked in water at room temperature for 24 h and then steamed until it was just beginning to be tender and intact. After cooling to room temperature, 2% (*w*/*w*) Jinhuangtian Jiuqu and 1.2% (*w*/*w*) Changleshao Jiuqu was each added to 250 g of steamed rice each and stirred evenly, and then it was transferred to the fermentation jar for scarification at 32 ± 1 °C for 24 h. Finally, 300 mL of sterile water was added to the fermentation jar for fermentation at 28 ± 1 °C for 15 days. Three biological replicates were performed and samples were collected daily from day 0 to day 15, and stored at −80 °C for further analysis.

### 2.2. Analysis of the Content of Higher Alcohols

For the analysis of higher alcohols, 50 mL of fermentation samples and 50 mL of deionized water were distilled to collect 50 mL of distillates. A total of 9.9 mL of distilled solutions were mixed with 100 μL of 2% (*v*/*v*) amyl acetate (internal standard), and filtered through a 0.22 µm filter for gas chromatography (GC) analysis [6].

Higher alcohol contents were determined using an Agilent 7860 GC equipped with a DB-624UI capillary column (60 m × 0.32 mm × 1.8 μm) and an FID detector (Agilent Technologies, Santa Clara, CA, USA) based on our lab optimization. The chromatographic conditions were based on the National Standard Method of Analysis for Baijiu (GB/T 10345-2022) [19] with some modifications. The detector and the injection temperature were 220 °C, and the injection volume was 1 μL. The carrier gas was hydrogen at 2 mL/min, and the split ratio was 37:1. The oven temperature was initially held at 30 °C for 6 min, increased to 40 °C at a heating rate of 2 °C/min and held for 2 min, then increased to 100 °C at a rate of 5 °C/min and held for 10 min, and finally raised to 200 °C at the rate of 10 °C/min and held for 10 min. The standard samples of n-propanol, isobutanol, isoamyl alcohol, 2-methyl-1-butanol and β-phenylethanol were used for qualitative and quantitative analysis of higher alcohols.

### 2.3. Analysis of the Precursors of Higher Alcohols

All fermentation samples were centrifuged at 12,000 rpm/min for 10 min prior to high-performance liquid chromatography (HPLC) equipped with a UV detector (Agilent Technologies, Santa Clara, CA, USA). For the analysis of organic acids, the supernatants were analyzed on an InfinityLab Poroshell 120 HILIC-Z column (4.6 mm × 100 mm, 2.7 μm, Agilent Technologies) [20]. The mobile phase was 0.03 mol/L diammonium hydrogen phosphate solution (pH 6.7)-acetonitrile solution (30:70, *v*/*v*) at a flow rate of 0.3 mL/min at 35 °C, and the detection wavelength was 215 nm. For ketoacid analysis, the supernatants were analyzed on Agilent ZORBAX SB-Aq column (4.6 mm × 250 mm, 5 μm) [21]. The mobile phase was 0.01 mol/L KH_2_PO_4_ solution (pH adjusted to 2.83 with phosphoric acid)-methanol (97:3, *v*/*v*) at a flow rate of 0.8 mL/min at 30 °C. The UV detection wavelength was 214 nm, and the injection volume was 10 μL. For amino acid analysis, the supernatants were analyzed on an Agilent AdvancedBio AAA column (4.6 mm × 100 mm, 2.7 μm) [22]. The mobile phase A was 10 mM Na_2_HPO_4_ and 10 mM Na_2_B_4_O_7_ (pH 8.2). The mobile phase B was acetonitrile: methanol: water (45:45:10, *v*/*v*/*v*), and the flow rate was 1.0 mL/min. The column temperature was 25 °C and the detection wavelength was 338 nm. Qualification and quantification of amino acids were determined by comparison of retention times and area under the curve with authentic standards.

### 2.4. Total DNA Extraction

Genomic DNA was extracted using HiPure Bacterial DNA Kits (Magen, China) according to the manufacturer‘s instructions. DNA quantification and purity were determined using Qubit (Thermo Fisher Scientific, Waltham, MA, USA) and Nanodrop (Thermo Fisher Scientific, Waltham, MA, USA), respectively.

### 2.5. Illumina Sequencing

Qualified genomic DNA was first fragmented by sonication to a size of 350 bp, and then end-repaired, A-tailed and adapter-ligated using the NEBNext^®^ Ultra™ DNA Library Prep Kit for Illumina^®^ (NEB, Ipswich, MA, USA) according to the manufacturer’s protocol. DNA fragments with length of 300–400 bp were enriched by PCR. The 50 μL reactions consisted of 23 μL adaptor ligated DNA fragments, 25 μL NEB Next High Fidelity 2X PCR Master Mix, 1 μL index primer and 1 μL universal PCR primer. The following thermocycling program was used: 98 °C 30 s; 98 °C 10 s, 65 °C 75 s, 72 °C 30 s, 12 cycles; 72 °C 5 min; 4 °C forever. The PCR products were purified using the AMPure XP system (Beckman Coulter, Brea, CA, USA), and libraries were analyzed for size distribution using the 2100 Bioanalyzer (Agilent, Santa Clara, CA, USA) and quantified by real-time PCR. The libraries were sequenced on an Illumina HiSeq X Ten sequencing platform at Guangzhou Gene Denovo Co., Ltd. (Guangzhou, China). Raw metagenomic data were submitted to the National Center for Biotechnology Information (NCBI) Sequence Read Archive (SRA) database (accession number PRJNA738725).

### 2.6. Bioinformatic Analysis

Raw sequencing data from the Illumina platform were filtered using FASTP (version 0.18.0) [23] according to the following standards: (1) remove reads with ≥10% unidentified nucleotides (N); (2) remove reads with ≥50% bases with Phred quality scores ≤ 20; (3) remove reads aligned to the barcode adapter. After filtering, the resulting clean reads were used for genome assembly.

Clean reads from each sample were individually assembled using MEGAHIT (version 1.1.2) [24], stepping over a k-mer range of 21 to 99 to generate a sample-derived assembly. Genes were predicted from the final assembly contigs (>500 bp) using MetaGeneMark (version 3.38) [25]. The predicted genes ≥300 bp in length from all samples were pooled and combined based on ≥95% identity and 90% reads coverage using CD-HIT (version 4.6) [26] to reduce the number of redundant genes for the downstream assembly step. Reads were realigned to the predicted gene using Bowtie (version 2.2.5) [27] to count the number of reads. The final gene catalog was generated from non-redundant genes with gene read counts > 2.

### 2.7. Function Annotations

The unigenes were annotated using DIAMOND by alignment with those deposited in various protein databases, including NCBI non-redundant protein database (NR), Kyoto Encyclopedia of Genes and Genomes (KEGG), Evolutionary Genealogy of Genes: Nonsupervised Orthologous Groups (eggNOG). Additional annotation was performed using the following databases: Carbohydrate-Active Enzymes (CAZy), Pathogen Host Interactions (PHI), Virulence Factors of Pathogenic Bacteria (VFDB) and Comprehensive Antibiotic Resistance Database (CARD).

### 2.8. Construction of the Higher Alcohols Metabolic Network

According to the KEGG database and existing literature, the metabolic pathways of major higher alcohols and the related enzymes involved in the metabolic pathways were sorted out. By comparing the functional gene with NR or KEGG database, the microorganism involved in higher alcohol metabolism was determined. Specifically, if a gene ID from the microorganism was annotated as an enzyme-encoding gene, the enzyme was associated with the microorganism. For example, if the unigene 246,148 was annotated as *Rhizopus microsporus* from the NR database and EC 2.6.1.66 from the KEGG database, the association was established between EC 2.6.1.66 and *R. microsporus*.

### 2.9. Statistical Analysis

Data were expressed as mean ± standard deviation (SD). Data were subjected to one-way ANOVA analysis using IBM SPSS version 22. Significant differences were determined by Duncan’s analysis (*p* < 0.05). Pearson’s correlation distance was performed using Origin 2019 (OriginLab Co., Northampton, MA, USA). Circular layout plots of species or functional gene abundance were plotted using circos (version 0.693) [28].

## 3. Results and Discussion

### 3.1. Changes in Higher Alcohols during Rice-Flavor Baijiu Fermentation

**Figure 2:**
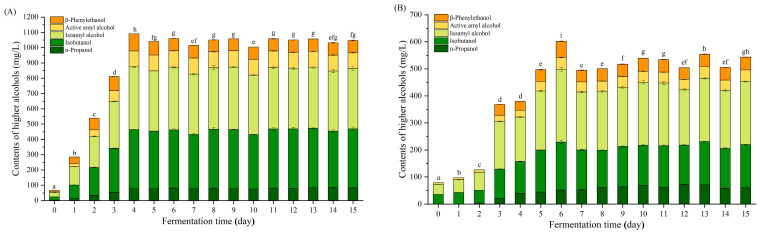
Changes in higher alcohols of JM and CM during fermentation. JM (**A**) and CM (**B**) were fermented by Jinhuangtian Jiuqufrom Heyuan Jinhuangtian Liquor Co., Ltd. and Changleshao Jiuqu from Guangdong Changleshao Co., Ltd., respectively (mean ± SD, *n* = 3). Columns with different letters indicate significant differences (*p* < 0.05).

The contents of higher alcohols in two kinds of fermentation materials were determined dynamically. As shown in Figure 2, the total higher alcohol content in JM at the beginning of fermentation was 66.19 mg/L, which was lower than that in CM (78.38 mg/L). However, the total content of JM increased rapidly, which was about three times that of CM on the first day, even four times that of CM on the second day, and maintained the large difference until the end. In general, the total content of higher alcohols in JM reached the maximum of 1091.03 mg/L on the fourth day, followed by a slight decrease, and then remained stable (Figure 2). A similar trend of changes was observed in the total higher alcohol content of CM, although the maximum was reached on the sixth day (601.42 mg/L). The most abundant higher alcohol in JM and CM was isoamyl alcohol, followed by isobutanol, two of which accounted for more than 70% throughout the fermentation process (Figure 2).

Isobutanol, isoamyl alcohol, n-propanol, active amyl alcohol and β-phenylethanol were the most abundant higher alcohols in Baijiu [2,5]. At the beginning of fermentation (day 0), the presence of higher alcohols (especially isobutanol, isoamyl alcohol and active amyl alcohol) in both JM and CM indicated that they were generated from the saccharification stage, which was similar to the visible higher alcohols at day 0 from another study on rice-flavor Baijiu [2]. In general, most of the higher alcohols were accumulated rapidly to the maximum during fermentation (Figure 2), while isoamyl alcohol and β-phenylethanol showed a decreasing trend in the first 2 days in another study [2]; these differences could be explained by the diverse microbial community and their metabolites.

### 3.2. Changes in Precursors of Higher Alcohols

**Figure 3:**
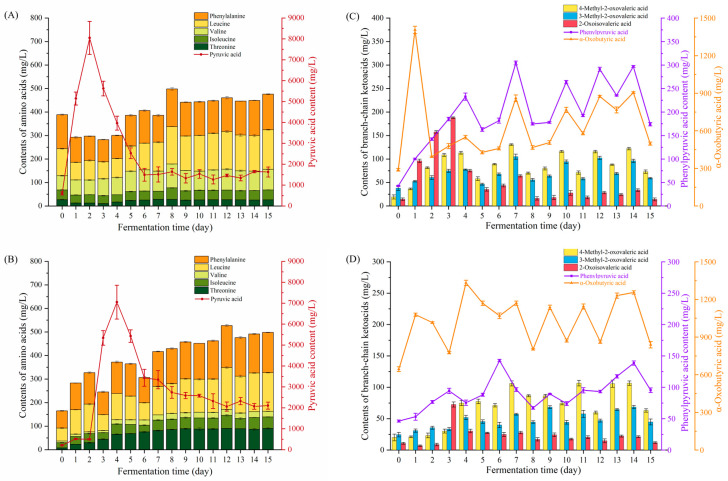
Changes in higher alcohol precursors of JM and CM during fermentation (mean ± SD, *n* = 3). The contents of amino acids and pyruvic acid were shown in JM (**A**) and CM (**B**), and the ketoacids were shown in JM (**C**) and CM (**D**).

The profiles of higher alcohol precursors (including amino acids, pyruvic acids and ketoacids) in two types of fermentation materials were presented as shown in Figure 3. Five amino acids (phenylalanine, leucine, valine, isoleucine and threonine) associated with higher alcohol metabolism were identified in JM and CM during fermentation (Figure 3A,B). Phenylalanine and isoleucine were much higher than others, accounting for more than half of the total amino acids in the two fermentation materials. In JM (Figure 3A), most of the amino acids showed a decreasing trend on the first day of fermentation except valine, which remained stable during the first two days. The highest total amino acid content of JM was observed on day 8 (545.67 mg/mL), and the lowest one was observed on day 3 (294.07 mg/mL). As for CM, the total amino acid content gradually increased and peaked on day 12 (567.25 mg/mL), except for the short-lived decrease on days 3, 6 and 13, which could be explained by the similar trend of phenylalanine, leucine and isoleucine (Figure 3B). The main differences between JM and CM were the changes that occurred on the first day of fermentation. In CM, five amino acids increased significantly from day 0 to day 1, which was similar to the increasing trend of amino acids in Shaoxing mechanized *huangjiu* during fermentation [29]. The biosynthetic pathways of these five amino acids could produce 𝛼-keto-acids, which could be converted into the corresponding higher alcohols [30]. Therefore, not only the microbiota, but also the amino acid composition could influence the composition and content of higher alcohols.

The initial pyruvic acid content of JM was 603.38 mg/L, which was 2.8 times higher than that of CM (Figure 3A,B). The pyruvic acid content of JM increased rapidly, reaching the highest value of 8051.50 mg/L on the second day, then decreased rapidly and remained stable from day 7 to the end of fermentation. In contrast, the pyruvic acid content of CM showed a huge increase from day 2 to day 4, with the maximum value of 7049.96 mg/L. The rapid increase and decrease in pyruvate content in the early stage of fermentation may be attributed to the conversion of glucose to pyruvate by microorganisms through glycolysis, and then pyruvate is rapidly consumed as a precursor of various metabolites [31,32].

The levels of other 𝛼-ketoacids in JM and CM varied significantly during fermentation (Figure 3C,D). In JM (Figure 3C), the concentration of α-oxobutyric acid was the highest, which increased rapidly to a maximum of 1405 mg/L on day 1, and then varied between approximately 400 and 1000 mg/L. The content of 2-oxoisovaleric acid peaked on day 3 (187.97 mg/L), followed by fluctuations in the range of 14.07–75.07 mg/L. The changes of phenylpyruvic acid, 4-methyl-2-oxovaleric acid and 3-methyl-2-oxovaleric acid were similar, fluctuating during the fermentation process and increasing to 173.98, 73.03 and 59.04 mg/mL, respectively, on day 15. Similarly to JM, α-oxobutyric acid was the most abundant ketoacid in CM (Figure 3D). Most of the 𝛼-ketoacid contents in CM witnessed an increase with a constant fluctuation, except for 2-oxoisovaleric acid, which peaked on day 3 and decreased to the initial values. The concentration of ketoacids in JM and CM during fermentation indicated that ketoacids were continuously synthesized, and the regular fluctuation of ketoacid contents suggested that ketoacids were consumed as intermediates in the process of continuous fermentation [33] or catalyzed to higher alcohols by the higher activity of decarboxylase and dehydrogenase [34].

It is worth noting that the differentiation of higher alcohol precursors between the two fermentation materials was not only in the concentration of each precursor, which led to the differences in higher alcohol contents in JM and CM. However, the change of higher alcohol precursors could not effectively clarify the reason for the differences of higher alcohols in the two mashes, so the role of microflora in JM and CM was further analyzed.

### 3.3. Microbiota Dynamics and Species Diversity Analysis during Rice-Flavor Baijiu Fermentation

In order to shed light on the genetic information of the microbial community during the fermentation of rice-flavor Baijiu, the microbial community of the JM and CM samples at different fermentation stages was analyzed by metagenomic sequencing technology. The raw data from the Illumina platform were 168.08 Gbp. To improve the accuracy of the subsequent analysis, the raw data were filtered to obtain clean data, which were 167.41 Gbp. The average percentage of clean bases with a correct recognition rate greater than 99% (Q20) was 97.08%, and the average percentage of clean bases with a correct recognition rate greater than 99.9% (Q30) was 91.87% (Table S1), suggesting the accuracy of the sequencing results. The average coverage and depth of sequencing were 89.16% and 59.94%, respectively.

**Figure 4:**
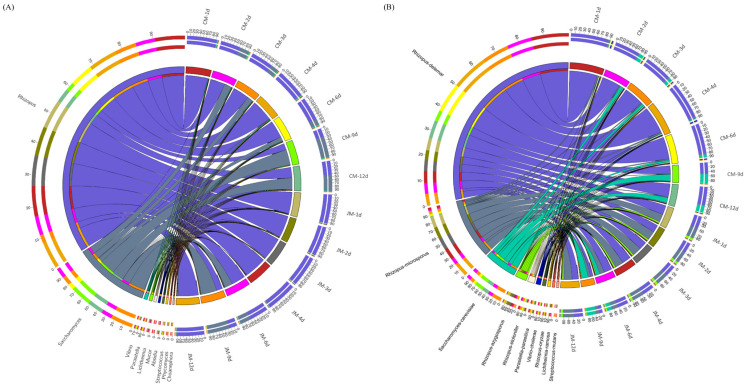
Distribution of the top 10 microbial taxa of JM and CM at the genus (**A**) and species (**B**) levels.

As shown in Figure 4, the dominant microorganisms (>0.05% abundance) at the genus level were *Rhizopus*, *Saccharomyces*, *Parasitella*, *Absidia*, *Lichtheimia* and *Bacillus*. Among them, *Rhizopus* and *Saccharomyces* dominated the microbial community in rice-flavor Baijiu fermentation samples, similar to the results reported by Hu et al. [2]. *Saccharomyces* belongs to *Ascomycota*, the relative abundance of which was overall higher in CM than in JM. As shown in Figure 4B, the relative abundance of *S. cerevisiae* in CM was higher than that in JM for most of the fermentation stage, except for day 6 and day 9, when *S. cerevisiae* increased substantially to peak at day 9 in CM and JM. Furthermore, another rapid increase in the relative abundance of *S. cerevisiae* was observed at day 2 in CM, which could explain the accumulation of higher alcohols in the early stage of fermentation. *Saccharomyces*, especially *S. cerevisiae*, is not only the main producer of ethanol, but also has an important influence on the synthesis and metabolism of higher alcohols during Baijiu fermentation [35,36].

*Rhizopus* belongs to *Mucoromycota*, whose relative abundance in CM was 93.11% on the first day of fermentation, decreased significantly on day 2, and then increased to 90.14% on day 4. From day 4 to day 9, the relative abundance decreased to the lowest at 26.03%, being the second dominant genus, and then increased slightly on day 12. The relative abundance of *Rhizopus* in JM presented a slow upward trend during the first four days of fermentation, after which it decreased to a minimum of 22.25% on day 9, and then increased rapidly to 84.27% on day 12. *Rhizopus* plays an important role in the production of enzymes such as amylase and glucoamylase for the saccharification and fermentation of Baijiu [37]. In addition, the higher relative abundance of *Rhizopus* was associated with the production of lactic acid and volatile compounds, which may affect the fermentation environment of other microbiota, such as *Saccharomyces* [38].

### 3.4. Distribution of Genes Associated with KEGG Pathways Related to Rice-Flavor Baijiu Fermentation

**Figure 5:**
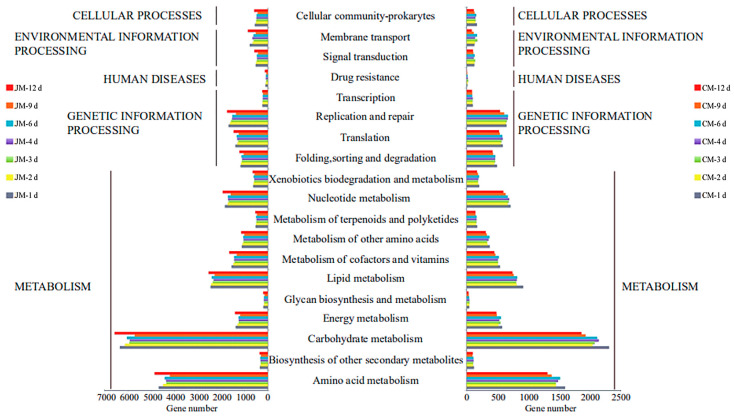
Functional diversity of KEGG metabolic pathways of JM and CM.

**Figure 6:**
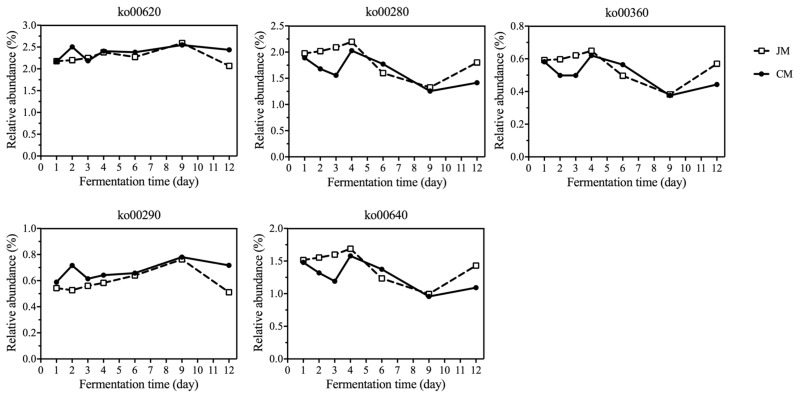
Relative abundance of carbohydrate metabolism related to higher alcohol metabolism in JM and CM during fermentation.

There were 59,593 (26.67%) unigenes in the gene catalog that were annotated within KEGG pathways. Five types of metabolic pathways were annotated according to the KEGG level 1 annotation, among which a large number of genes belonged to metabolism (Figure 5). Based on the level 2 annotation, the relative abundance of carbohydrate metabolism was the largest, followed by amino acid metabolism, indicating that microorganisms were enriched with genes involved in carbohydrates and amino acids during the fermentation process of rice-flavor Baijiu (Figure 5). In addition, among the level 3 pathways, ko00620 (pyruvate metabolism), ko00290 (valine, leucine and isoleucine biosynthesis), ko00280 (valine, leucine and isoleucine degradation), ko00640 (propanoate metabolism) and ko00360 (phenylalanine metabolism) were associated with higher alcohol synthesis. The variation trends of the relative abundance of ko00620 and ko00290 in CM and JM were similar to those of the other three pathways (Figure 6). As for JM, the relative abundance of genes involved in these pathways increased during the early fermentation stages (day 1 to day 4), suggesting that the microbiota in JM might be more active in higher alcohols synthesis.

### 3.5. Metabolic Pathways of Higher Alcohols and Microbial Distribution during Rice-Flavor Baijiu Fermentation

**Figure 7:**
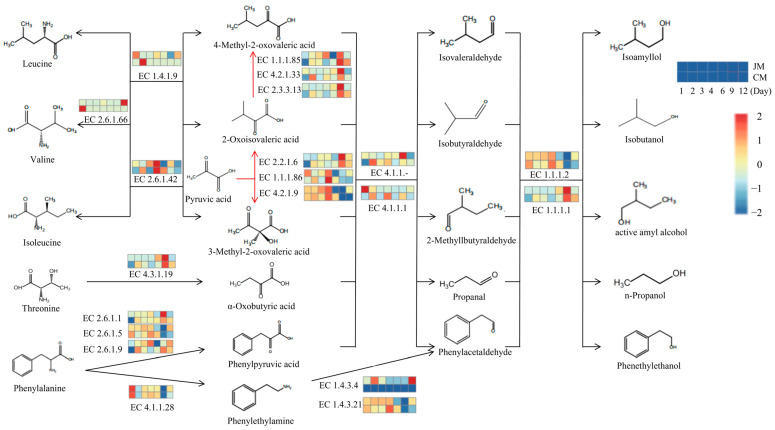
Inferred higher alcohol synthetic pathway and number of the annotated enzymes within the metagenomes. The numbers of the annotated enzymes are expressed in heat maps. The two rows represent CM and JM, and the seven columns represent the fermentation time points (1, 2, 3, 4, 6, 9 and 12 days) in each heat map.

Higher alcohols are by-products of Baijiu fermentation, and their content affects the taste and quality of Baijiu. The biosynthesis of higher alcohols includes the Ehrlich pathway for amino acid catabolism and the Harris pathway for sugar metabolism. In general, the variation in the abundance of genes involved in higher-alcohol-related pathways in JM and CM suggested that the synthesis of higher alcohols in Baijiu fermentation is extremely complex (Figure 7). Pyruvic acid is the precursor for the synthesis of valine, leucine and isoleucine, known as branched-chain amino acids. In the Ehrlich pathway, these amino acids are converted to α-ketoacids (2-oxoisovaleric acid, 4-methyl-2-oxovaleric acid and 3-methyl-2-oxovaleric acid) through the pathway of ko00280 catalyzed by branched-chain amino acid aminotransferase (EC 2.6.1.42), and then decarboxylated and dehydrogenated to generate corresponding higher alcohols (isobutanol, isoamyl alcohol and active amyl alcohol) by decarboxylases (EC 4.1.1.-; 4,1,1,1) and dehydrogenase (EC 1.1.1.2; 1,1,1,1). Pyruvic acid is also catalyzed to (s)-2-acetolate (AL) by alpha-acetolactate synthase (EC 2.2.1.6), sequentially to 2-oxoisovaleric acid under the catalysis of ketol-acid reductoisomerase (EC 1.1.1.86) and dihydroxy-acid dehydratase (EC 4.2.1.9), and then converted to 4-methyl-2-oxovaleric acid under the stepwise catalysis of 2-isopropylmalate synthase (EC 2.3.3.13), 3-isopropylmalate dehydratase (EC 4.2.1.33) and 3-isopropylmalate dehydrogenase (EC 1.1.1.8). However, the decreased pyruvic acid levels were observed later than the rapid accumulation of higher alcohols, which may indicate that the Ehrlich pathway was mainly responsible for the synthesis of higher alcohols during the early fermentation of rice-flavor Baijiu. In addition, valine pyruvate aminotransferase (EC 2.6.1.66) can catalyze the conversion of valine to 2-oxoisovaleric acid. Leucine dehydrogenase (EC 1.4.1.9) is another primary enzyme that catalyzes the redox reaction between branched-chain amino acids and the corresponding ketoacids. In this study, n-propanol biosynthesis was annotated only by the immediate precursor of threonine, which was catalyzed and deaminated by L-serine deaminase (EC 4.3.1.19) to produce 𝛼-oxobutyric acid, then decarboxylated and dehydrogenated to produce n-propanol. For phenylethanol, two pathways were observed in JM and CM. In the pathway of phenylpyruvic acid synthesis, phenylalanine was catalyzed by aspartate aminotransferase (EC 2.6.1.1), tyrosine aminotransferase (EC 2.6.1.5) and histidine phosphate aminotransferase (EC 2.6.1.9) to phenylpyruvic acid. Phenylalanine can also be catalyzed by aromatic L-amino acid decarboxylase (EC 4.1.1.28) to form phenylethylamine and then oxidized by monoamine oxidase (EC 1.4.3.4) or primary amine oxidase (EC 1.4.3.21) to phenylacetaldehyde.

**Figure 8:**
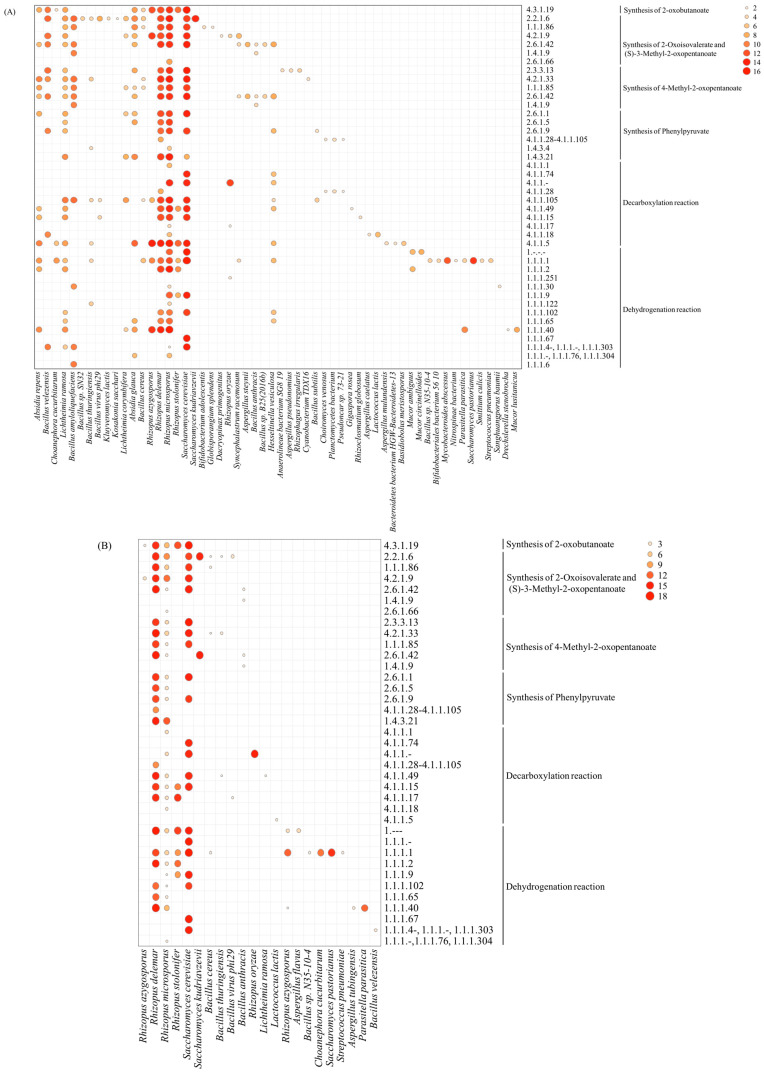
The correlation between microorganisms and enzymes involved in different metabolic pathways involved in the production of higher alcohols from JM (**A**) and CM (**B**).

The relationship between microorganisms at the species level and enzymes involved in higher alcohol metabolic pathways is shown in Figure 8. In general, *Rhizopus* and *Saccharomyces* were most involved in most processes of higher alcohol biosynthesis in JM and CM. However, there were more microorganisms involved in the synthesis of higher alcohols in JM than in CM. Besides *Rhizopus* and *Saccharomyces*, there were other functional microorganisms involved in the biosynthesis of higher alcohols in JM, especially *Absidia*, *Lichtheimia* and *Bacillus*, which could be involved in the synthesis of immediate precursors, including 𝛼-oxobutyric acid, α-ketoacids and phenylpyruvic acid (Figure 3). In addition, *Hesseltinella* was observed in JM as a producer of aminotransferase, decarboxylases, and dehydrogenase in the Ehrlich pathway. It was found that the dominant microorganisms annotated with decarboxylases (EC 4.1.1.-) in CM at the species level were *R. oryzae* and *S. cerevisiae*, and pyruvate decarboxylase (EC 4.1.1.1) was annotated only in *Rhizopus microsporus*. Besides *R. oryzae* and *S. cerevisiae*, the dominant microorganisms annotated by EC 4.1.1.- in JM included *R. microsporus* and *H. viculosa*, and EC 4.1.1.1 was also annotated only in *R. microsporus*. As shown in Figure 4, the relative abundance of *R. microsporus* was higher in JM than in CM, which may be one of the reasons for the higher content of higher alcohols in JM than in CM. As expected for *Saccharomyces*, *Rhizopus* has been reported to produce n-propanol, 3-methylbutanol and other volatiles during fermentation [39,40]. Furthermore, the abundance of *Rhizopus* in Xiaoqu was more important than that in Daqu because it was closely related to starch hydrolysis, alcohol fermentation and flavor [41]. Therefore, the comprehensive analysis between microbiota, functional genes, and higher-alcohol-related compounds indicated that *R. delemar*, *R. microsporus*, and *S. cerevisiae* might be the most important functional microorganisms for the synthesis of higher alcohols in rice-flavor Baijiu.

## 4. Conclusions

In the present study, metagenomic and metabolic profiling were performed to explore the correlation between the microbiota and higher alcohols along with precursors during rice-flavor Baijiu fermentation. Significantly higher levels of higher alcohols were observed in JM than in CM during fermentation, which could be attributed to their rapid growth during the early stage of fermentation. Similarly to the changes in higher alcohols, variations in their corresponding precursors were also observed during fermentation. Based on the metagenomic analysis, *Rhizopus* and *Saccharomyces* were the dominant microorganisms for rice-flavor Baijiu brewing. *S. cerevisiae* and *R. microsporus* were involved in the whole process for higher alcohol formation in both JM and CM; however, the relative abundance of *R. microsporus* was much higher in JM than in CM, which might contribute to the higher alcohol formation in JM.

## Data Availability

The data presented in this study are available on request from the corresponding author.

## Supplementary Materials

The following supporting information can be downloaded at: https://www.mdpi.com/article/10.3390/foods12142720/s1. Figure S1: Profiles of higher alcohols in nine types of fermented mashes inoculated with different starters. Table S1: Sequencing information and data quality control of metagenomic analysis.
