# Supplementary material for: Characterization and Correlation of Microbiota and Higher Alcohols Based on Metagenomic and Metabolite Profiling during Rice-Flavor Baijiu Fermentation
Source: Foods. 2023 Jul 16;12(14):2720. doi: 10.3390/foods12142720 (PMC10379614; doi:10.3390/foods12142720)
Supplement: Supplementary file 1 [file foods-12-02720-s001.zip › foods-2458764-supplementary.pdf]

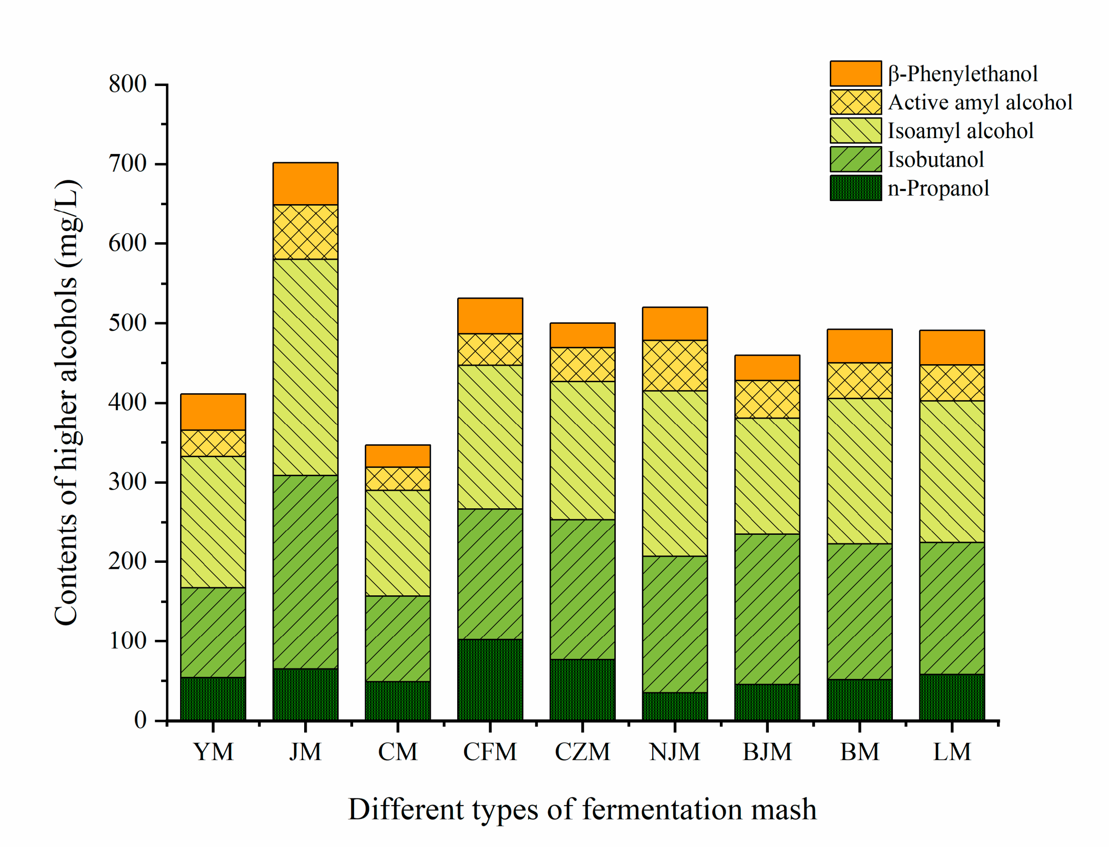

Figure S1. Profiles of higher alcohols in nine types of fermented mashes inoculated with different starters. YM means fermentation mash inoculated with Yingjinqian Jiuqu from Guangzhou Eagle-Coin Food Group Co., Ltd. (Guangzhou, China). JM means fermentation mash inoculated with Jinhuangtian Jiuqu from Heyuan Jinhuangtian Liquor Co., Ltd. (Heyuan, Guangdong, China). CM means fermentation mash inoculated with Changleshao Jiuqu (rice flour) from Guangdong Changleshao Co., Ltd. (Meizhou, Guangdong, China). CFM means fermentation mash inoculated with Changleshao Jiuqu (bran) from Guangdong Changleshao Co., Ltd. (Meizhou, Guangdong, China). CZM means fermentation mash inoculated with ester producing Xiaoqu from Guangzhou Eagle-Coin Food Group Co., Ltd. (Guangzhou, China). NJM means fermentation mash inoculated with liquor-producing starter from Angel Yeast Co., Ltd. (Yichang, Hubei, China). BJM means fermentation mash inoculated with Baijiu Qu from Angel Yeast Co., Ltd. (Yichang, Hubei, China). BM means fermentation mash inoculated with Bazhenniang Jiuqu from Meizhou Bazhenniang Liquor Co., Ltd. (Meizhou, Guangdong, China). LM means fermentation mash inoculated with Laobachi Jiuqu from Guangdong Hemixiang Liquor Co., Ltd. (Meizhou, Guangdong, China).

Table S1: Sequencing information and data quality control of metagenomic analysis.

Samples	Clean Data (Gbp)	Clean Q20 (%)	Clean Q30 (%)	Coverage (%)	Depth	Contig number	Average length (bp)	N50 Length (bp)	unigenes
CM-1d-1	6.78	97.01	91.64	86.79	92.86	14287	3199	13303	63555
CM-1d-2	7.09	96.92	91.44	86.86	107.74				59917
CM-2d-1	5.96	96.87	91.4	92.79	65.96	10771	4952	19352	60198
CM-2d-2	6.35	97.21	92.02	90.5	111.14				59484
CM-3d-1	5.95	96.65	90.86	93.46	79.45	12090	4633	17990	62969
CM-3d-2	5.99	96.7	90.91	93.49	68.79				62471
CM-4d-1	7.28	97.3	92.17	91.71	113.82	11006	4817	16985	61662
CM-4d-2	6.13	97.05	91.64	91.13	98.43				61596
CM-6d-1	6.05	96.81	91.14	92.82	82.11	10848	4907	18939	60892
CM-6d-2	7.12	97.14	91.88	91.65	104.62				62641
CM-9d-1	7.06	97.14	91.82	92.83	81.78	10238	5104	16669	59569
CM-9d-2	6.32	97.24	91.95	93.71	79.5				55619
CM-12d-1	7.02	97.21	92.04	94.85	97.66	10147	5170	18406	58037
CM-12d-2	7.31	96.91	91.36	94.73	93.6				57683
JM-1d-1	4.54	97.5	92.97	84.6	24.59	112436	1430	2423	138360
JM-1d-2	5.51	96.27	90.5	87.59	22.65				168072
JM-2d-1	5.12	97.25	92.5	86.76	24.65	50853	2547	9291	153958
JM-2d-2	4.68	97.34	92.62	86.87	25.37				138128
JM-3d-1	5.11	97.24	92.44	85.83	28.33	49128	2395	6712	135925
JM-3d-2	5.03	96.1	90.09	86.58	27.97				137871

JM-4d-1	5.09	97.28	92.51	83.49	31.82	39294	2459	13659	124136
JM-4d-2	4.74	97.18	92.27	84.24	29.43				126475
JM-6d-1	7.07	97.47	92.87	88.39	35.89	42051	2877	12964	135983
JM-6d-2	6.84	97.44	92.44	88.37	37.71				136512
JM-9d-1	6.04	97.33	92.18	84.75	31.99	34126	3195	6786	109893
JM-9d-2	6.79	97.16	91.82	88.25	35.94				126526
JM-12d-1	4.54	97.26	92.61	86.24	21.29	65808	2084	7607	159506
JM-12d-2	4.57	97.19	92.36	87.29	23.36				151194
